# Amplicon-Dependent *CCNE1* Expression Is Critical for Clonogenic Survival after Cisplatin Treatment and Is Correlated with 20q11 Gain in Ovarian Cancer

**DOI:** 10.1371/journal.pone.0015498

**Published:** 2010-11-12

**Authors:** Dariush Etemadmoghadam, Joshy George, Prue A. Cowin, Carleen Cullinane, Maya Kansara, Australian Ovarian Cancer Study Group, Kylie L. Gorringe, Gordon K. Smyth, David D. L. Bowtell

**Affiliations:** 1 Cancer Genomics Program, Peter MacCallum Cancer Centre, East Melbourne, Australia; 2 Department of Biochemistry, University of Melbourne, Parkville, Australia; 3 Translational Research Program, Peter MacCallum Cancer Centre, East Melbourne, Australia; 4 Department of Pathology, University of Melbourne, Parkville, Australia; 5 Bioinformatics Division, Walter and Eliza Hall Institute of Medical Research, Parkville, Australia; Chinese University of Hong Kong, China

## Abstract

Genomic amplification of 19q12 occurs in several cancer types including ovarian cancer where it is associated with primary treatment failure. We systematically attenuated expression of genes within the minimally defined 19q12 region in ovarian cell lines using short-interfering RNAs (siRNA) to identify driver oncogene(s) within the amplicon. Knockdown of *CCNE1* resulted in G1/S phase arrest, reduced cell viability and apoptosis only in amplification-carrying cells. Although *CCNE1* knockdown increased cisplatin resistance in short-term assays, clonogenic survival was inhibited after treatment. Gain of 20q11 was highly correlated with 19q12 amplification and spanned a 2.5 Mb region including *TPX2*, a centromeric protein required for mitotic spindle function. Expression of *TPX2* was highly correlated with gene amplification and with *CCNE1* expression in primary tumors. siRNA inhibition of *TPX2* reduced cell viability but this effect was not amplicon-dependent. These findings demonstrate that *CCNE1* is a key driver in the 19q12 amplicon required for survival and clonogenicity in cells with locus amplification. Co-amplification at 19q12 and 20q11 implies the presence of a cooperative mutational network. These observations have implications for the application of targeted therapies in *CCNE1* dependent ovarian cancers.

## Introduction

Advanced stage serous tumors account for the majority of invasive ovarian cancers and despite a generally good initial response to cytoreductive surgery and platinum-based chemotherapy, most women face a high risk of recurrence and poor long-term survival [1]. Platinum-based agents, such as cisplatin and carboplatin, are toxic to dividing cells due to the formation of DNA adducts that result in double strand breaks, activating DNA damage-mediated apoptotic signals [2]. Response to chemotherapy is, however, difficult to predict and there are currently no predictive biomarkers for serous ovarian cancers in clinical use. We have previously mapped a region of 19q12 amplification associated with treatment-resistant serous ovarian tumors by performing a genome-wide survey of copy number change [3]. These findings were consistent with previous reports of amplification being associated with poor overall survival [4], [5]. Similarly, recurrent amplification of 19q12 has been reported in a variety of cancers including esophageal [6], gastric [7], lung [8] and endometrial tumors [9].

The 19q12 amplification is a high-level focal amplification that targets a cluster of only several genes on chromosome 19. *CCNE1* (Cyclin E) has previously been suggested as the target of amplification in ovarian cancer [4], [10], [11], however a systematic analysis of known genes within the amplicon has not been performed. Furthermore, whilst *CCNE1* amplification likely provides an oncogenic stimulus through activation of the cell cycle, it is not obvious how it may contribute to primary chemotherapy resistance. For example, over-expression of *CCNE1 in vitro* renders ovarian cancer cells more sensitive to platinum agents, presumably due to increased proliferation [12]. It is possible that the biological consequence of 19q12 amplification is not limited to over-expression of *CCNE1*, and that other genes in the amplicon contribute to tumor growth or progression. Furthermore, other co-existing mutational events elsewhere in the cancer genome may cooperate or enhance the oncogenic effect of *CCNE1* over-expression.

We performed an siRNA knockdown screen of all annotated genes within and immediately flanking the 19q12 amplicon in ovarian cancer cell lines with or without regional amplification. We found *CCNE1* to be the only gene target within the amplicon that reduced cell viability in the amplicon-containing OVCAR-3 cell line after siRNA knockdown. *CCNE1* knockdown induced cell cycle arrest and apoptosis, while also impairing clonogenic survival after cisplatin treatment, despite increasing *in vitro* drug resistance in a short-term cytotoxicity assay. In a disease setting, these results suggest that treatment failure in *CCNE1* amplified tumors may relate to rapid repopulation of the tumor after chemotherapy and not cellular drug resistance specifically. We also found *TPX2* amplification and over-expression to be significantly correlated with *CCNE1* copy number status implying the presence of a cooperative mutational network between these genes.

## Results

### Focal amplification of 19q12 is common to various tumor types

**Figure 1:**
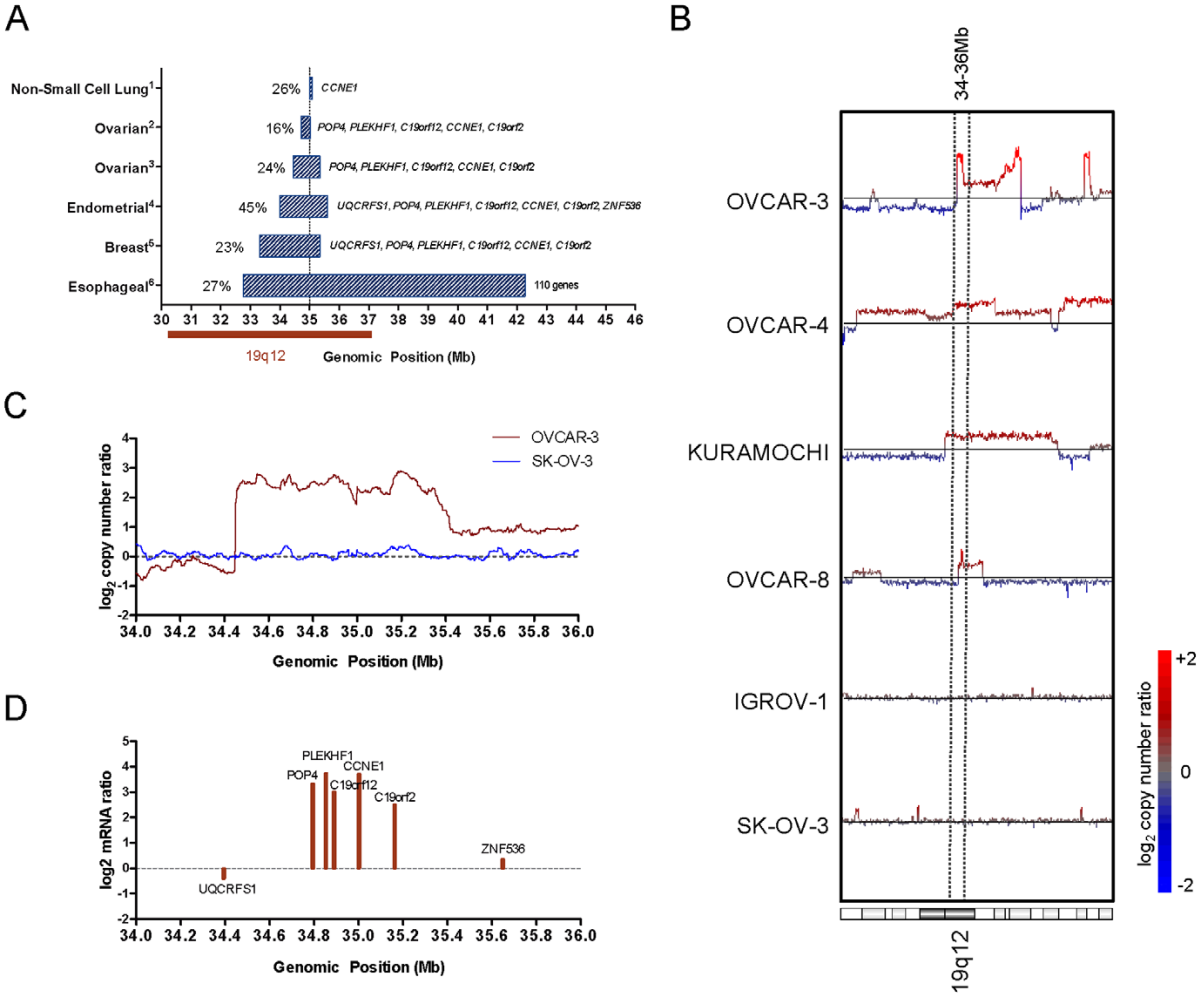
19q12 amplification in tumors and ovarian tumor cell lines. (A) Peak regions of amplification between 30–46 Mb on chromosome 19 in ^1^non-small cell [8], [13], [40], [41], [42]; ^2^ovarian [3], ^3^ [13]; ^4^endometrial [9]; ^5^breast [13], [43], [44]; and ^6^esophageal tumors [42]. Frequency of occurrence and genes present within peak boundaries indicated. (B) Affymetrix SNP 6.0 mapping microarray copy number of chromosome 19 in ovarian tumor cell lines and (C) between 34–36 Mb for OVCAR-3 and SK-OV-3 cell lines. Copy number shown is the average moving window of 20 markers mapped to Human March 2006 (hg18) genome assembly (source: Sanger Cancer Genome Project Archive). (D) Gene expression determined by qPCR in OVCAR-3 cells relative to SK-OV-3.

We first sought to compare the minimal region of chromosomal gain at 19q12 across multiple tumor types. We obtained data from SNP-based high-resolution copy number studies including 15 tumor types [9], [13] for a comparison with our findings [3] (Figure 1A). Minimal targeted regions of amplification were defined by GISTIC, an analysis tool that assesses the statistical significance of copy number events based on frequency and amplitude [14]. Significant amplification of 19q12 was present in one third of the cancer types analyzed. Of the tumor types with 19q12 amplification, approximately 25% of individual samples showed copy number gain, except for endometrial tumors where a higher frequency was observed (∼45%) [9]. Minimal amplicon boundaries were found to target a region less than 2 Mb in size, centered at approximately at 35.0 Mb on chromosome 19. In both ovarian tumor data sets analyzed [3], [13] the minimal mapped region of gain incorporated the same five genes (*POP4*, *PLEKHF1*, *C19orf12, CCNE1 and C19orf2*), with similar overlapping regions detected in endometrial and breast tumors. In contrast, the minimal region mapped in non-small cell lung tumors incorporated only *CCNE1* while a broad region was mapped in esophageal tumors (∼9.5 Mb), spanning 110 annotated genes. Copy number change of the 19q12 locus showed a degree of tumor specificity, in that the amplification was not seen in 10 other tumor types for which substantial data was available, including small cell lung, hepatocellular, colorectal and prostate cancer (data not shown). We also note that amplification of 19q12 has been identified by cDNA array-CGH analysis of gastric tumors [15], [16], however this tumor type was not included as our analysis was limited to high-resolution SNP copy number data.

To identify cell lines that were representative of primary tumors for functional studies we analyzed high-resolution SNP copy number data for 22 ovarian cancer cell lines at chromosome 19q12 (Sanger Cancer Genome Project Archive) and identified seven cell lines (OVCAR-3, OVCAR-4, Kuramochi, RMG-I, Caov-4, EFO-21, OVCAR-8) that had overlapping amplification at 19q12 (Figure 1B and Figure S1). Of the seven cell lines, OVCAR-3 contained a focal, high-level amplification that best recapitulated data from primary ovarian tumors (Figure 1C). Quantitative-PCR (qPCR) demonstrated that the five genes within the region of high-level amplification in OVCAR-3 (*POP4*, *PLEKHF1*, *C19orf12*, *CCNE1 and C19orf2*), but not flanking genes (*UQCRFS1* and *ZNF536*), were over-expressed relative to the SK-OV-3 control cell line lacking 19q12 amplification (Figure 1D). Of the five genes, *PLEKHF1* and *CCNE1* showed the highest expression. The 19q12 amplicon can therefore be mapped to a region spanning 34.4–35.4 of chromosome 19 and involving 5 annotated genes, each of which is over-expressed in OVCAR-3.

### Cells lines with amplification at 19q12 are specifically sensitive to *CCNE1* knockdown

**Figure 2:**
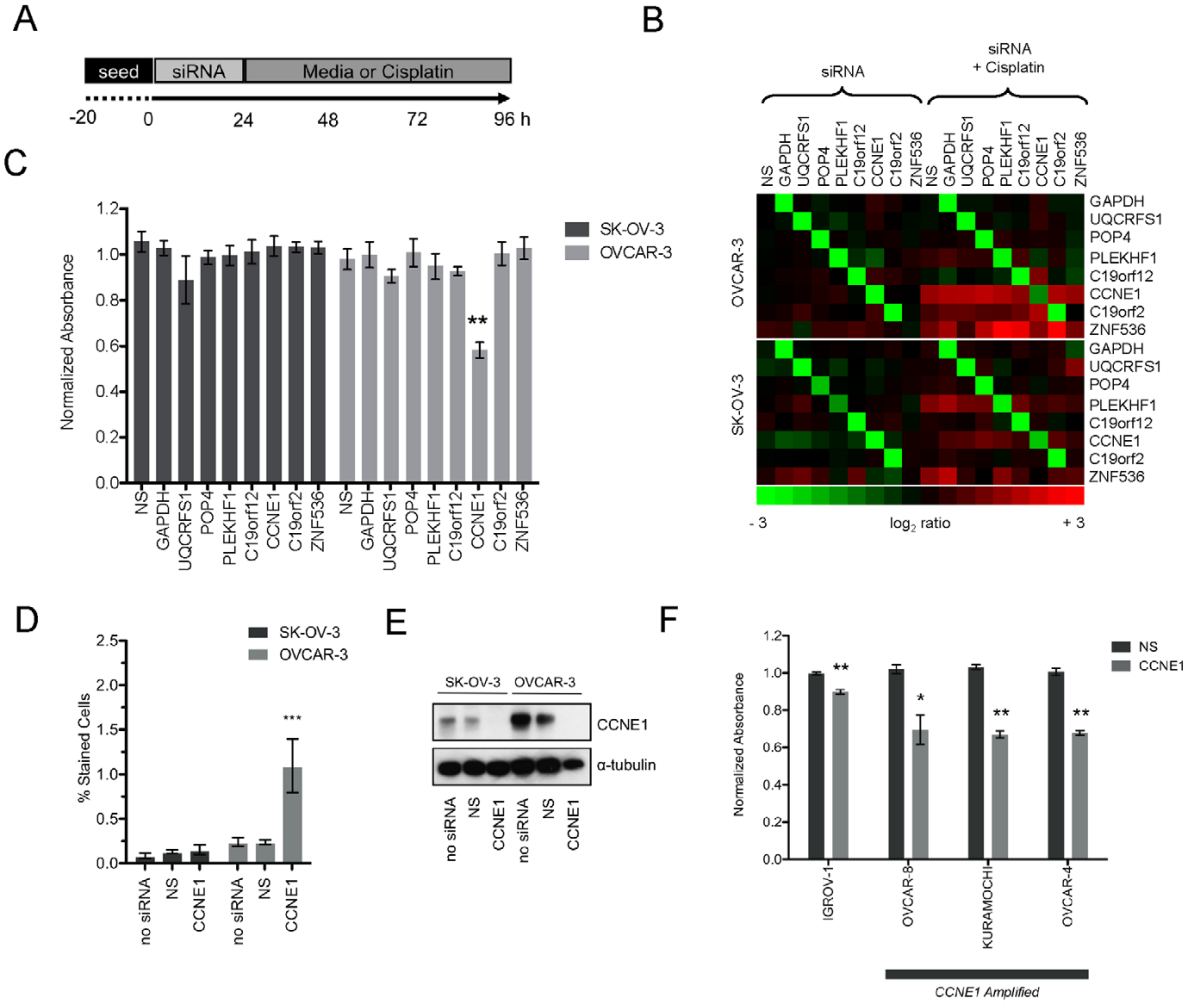
siRNA mediated knock-down of 19q12 genes in ovarian tumor cell lines. (A) Experimental schematic for combined siRNA transfection and drug treatment. (B) qPCR heatmap showing log_2_ gene expression ratio to untransfected OVCAR-3 (top) and SK-OV-3 (bottom) cells for each siRNA (columns) at gene targets (rows) with or without cisplatin treatment 72 hours after transfection. (C) Cell viability normalized to no siRNA control cells after transfection with each siRNA. Statistical significance (t-test) calculated by comparison to non-silencing (NS) siRNA in the same cell line using data from three independent MTS assays performed with triplicate wells per condition. Average normalized absorbance at 490 nm and SEM plotted (n = 3), **p-value <0.01. (D) Proportion of apoptotic cells assessed by TUNEL staining in no siRNA control cells or after transfection with NS or *CCNE1* targeted siRNA. Data shown from three independent experiments with duplicate wells analyzed per condition. Average percentage of apoptotic cells and SEM plotted (n = 3). ***p-value <0.0001 (Chi squared) calculated by comparison of total cell counts between OVCAR-3 cells treated with a *CCNE1* siRNA and all other conditions. (E) CCNE1 protein expression by western blot to confirm siRNA-mediated *CCNE1* knockdown at experimental endpoint. (F) Cell viability in additional cell lines after transfection with *CCNE1* or non-silencing siRNAs normalized to each cell line with no siRNA added. Statistical significance (t-test) calculated by comparison to NS siRNA in the same cell line. Average normalized absorbance from MTS assay and SEM plotted (n = 3); *p-value <0.05, **p-value <0.01.

Short interfering RNA (siRNA) were used to knockdown the expression of the seven genes in and adjacent to the high-level 19q12 amplicon in OVCAR-3 and SK-OV-3 plus *GAPDH* and non-silencing (NS) controls. A schematic of the experimental design used for the combined siRNA knockdown strategy and subsequent drug treatment protocol is shown in Figure 2A. Transcript levels for all targeted genes were efficiently and specifically reduced up to 96 hours after siRNA transfection, with the exception of *ZNF536*, which flanked the region of amplification (Figure 2B). By interrogating data obtained from an earlier study [17] we found *ZNF536* expression to be low or absent in primary serous ovarian tumors, which may explain an unobservable reduction in gene expression (data not shown). Gene expression was also monitored in the presence of cisplatin, in preparation for functional experiments. *PLEKHF1*, *CCNE1*, *C19orf2* and *ZNF536* were slightly up-regulated by cisplatin treatment in one or both cell lines however siRNA knockdown was still effective (Figure 2B).

Of the genes tested, only *CCNE1* knockdown showed a significant reduction in cell viability in OVCAR-3 (to approximately 60% of NS control cells; p<0.01; Figure 2C). *CCNE1* knockdown had no effect on SK-OV-3 cells. Given its role in G1/S transition, we expected that depletion of Cyclin E1 protein would induce G1 arrest (see below) and result in an increase of apoptotic cells. TUNEL staining of untreated cells was comparable (SK-OV-3, 0.1%; OVCAR-3, 0.2%) (Figure 2D), while *CCNE1* knockdown resulted in a significant increase in apoptosis only in OVCAR-3 (p<0.0001). Reduction in protein abundance was also validated in both cell lines after gene knockdown (Figure 2E).

Having identified specific sensitivity of OVCAR-3 to *CCNE1* knockdown, we aimed to validate this finding in independent cell lines and determine whether the observed effect was amplicon dependent. We therefore broadened the analysis to include an additional three cell lines with amplification at 19q12 (OVCAR-4, Kuramochi and OVCAR-8) and a further unamplified line (IGROV-1). A statistically significant correlation between the copy number and gene expression of *CCNE1* was found across all lines. However, OVCAR-8 did not show increased *CCNE1* expression relative to gene amplification (Figure S2 A). *CCNE1* expression and Cyclin E1 protein levels were efficiently reduced in each line relative to base-line expression by siRNA-mediated knockdown (Figure S2 B and C). As observed in OVCAR-3, *CCNE1* knockdown specifically reduced viability in the additional lines with 19q12 amplification, and only marginally in the unamplified line IGROV-1 (Figure 2F). Thus ovarian tumor cells with amplification at 19q12 are specifically sensitive to depletion of Cyclin E1, compared with unamplified lines.

### *CCNE1* knockdown reduces acute sensitivity to cisplatin

**Figure 3:**
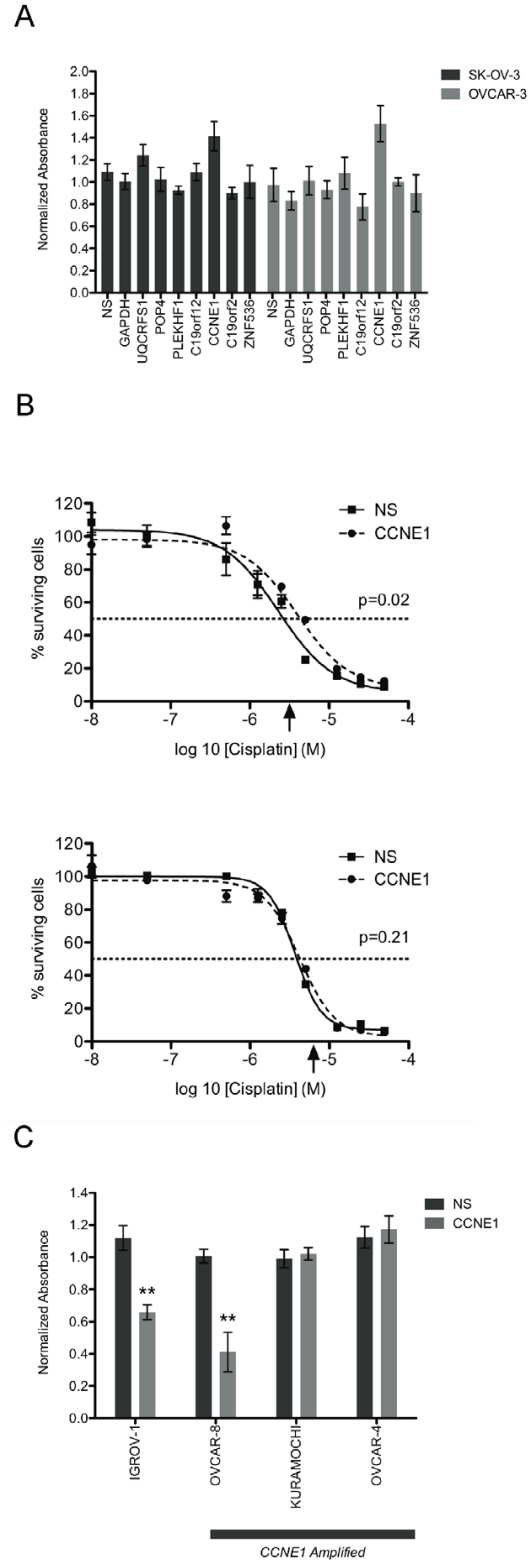
Combined siRNA knock-down and cisplatin treatment in ovarian tumor cell lines. (A) Cell viability after transfection with individual siRNAs and cisplatin treatment normalized to cisplatin-treated control cells without siRNA. Cisplatin dose of 3 µM or 6 µM was used for OVCAR-3 and SK-OV-3 cells respectively. Average normalized absorbance (490 nm) from three independent MTS assays (triplicate wells per condition) and SEM plotted (n = 3). (B) Cisplatin dose response after transfection with *CCNE1* or non-silencing siRNA in OVCAR-3 and SK-OV-3 cell lines. Arrow indicates drug treatment dose used in initial screen; p-value indicates significance of difference between fitted curves. Average normalized MTS assay absorbance to cells without cisplatin treatment, SEM and four-parameter fitted Hill slope plotted (n = 3 for each drug concentration). (D) Cell viability after transfection with *CCNE1* or NS siRNAs and cisplatin treatment normalized to cisplatin-treated no siRNA control cells for each cell line. Statistical significance calculated by comparison to NS siRNA, cisplatin-treated cells in the same cell line. Average normalized absorbance from MTS assay and SEM plotted (n = 3). See Table S4 for cisplatin treatment doses.

Given the association of 19q12 amplification with primary treatment failure [3] and poor outcome [4], [5] we sought to explore the effect of gene knockdown on drug sensitivity. Although our analysis had identified a specific dependency on *CCNE1* in amplified lines, we first re-assessed all seven amplicon-associated genes for impact on chemotherapy response in OVCAR-3 and SK-OV-3 using a 72-hour cytotoxicity assay. Cells were treated at slightly above a pre-determined IC50 (see Methods S1) and viability measured. Knockdown of genes within the amplicon did not significantly impact on cisplatin sensitivity of either cell line. Unexpectedly, cisplatin-induced cytotoxicity in OVCAR-3 treated cells was attenuated by *CCNE1* inhibition (Figure 3A). We performed a dose-response analysis to characterize further the effect of cisplatin treatment after *CCNE1* knockdown. A statistically significant shift in the dose-response curve was observed in OVCAR-3 (p<0.05; Figure 3B) but not SK-OV-3 further demonstrating resistance of OVCAR-3 to cisplatin upon *CCNE1* inhibition. Consistent with this finding, cisplatin had no differential effect on cell viability in *CCNE1* knockdown cells as compared with siRNA controls in two of the three additional 19q12 amplified lines (Kuramochi and OVCAR-4) (Figure 3C). In contrast, gene knockdown enhanced the effect of cisplatin on cell viability in both cell lines with low baseline *CCNE1* expression (OVCAR-8 and the 19q12 unamplified control line IGROV-1).

**Figure 4:**
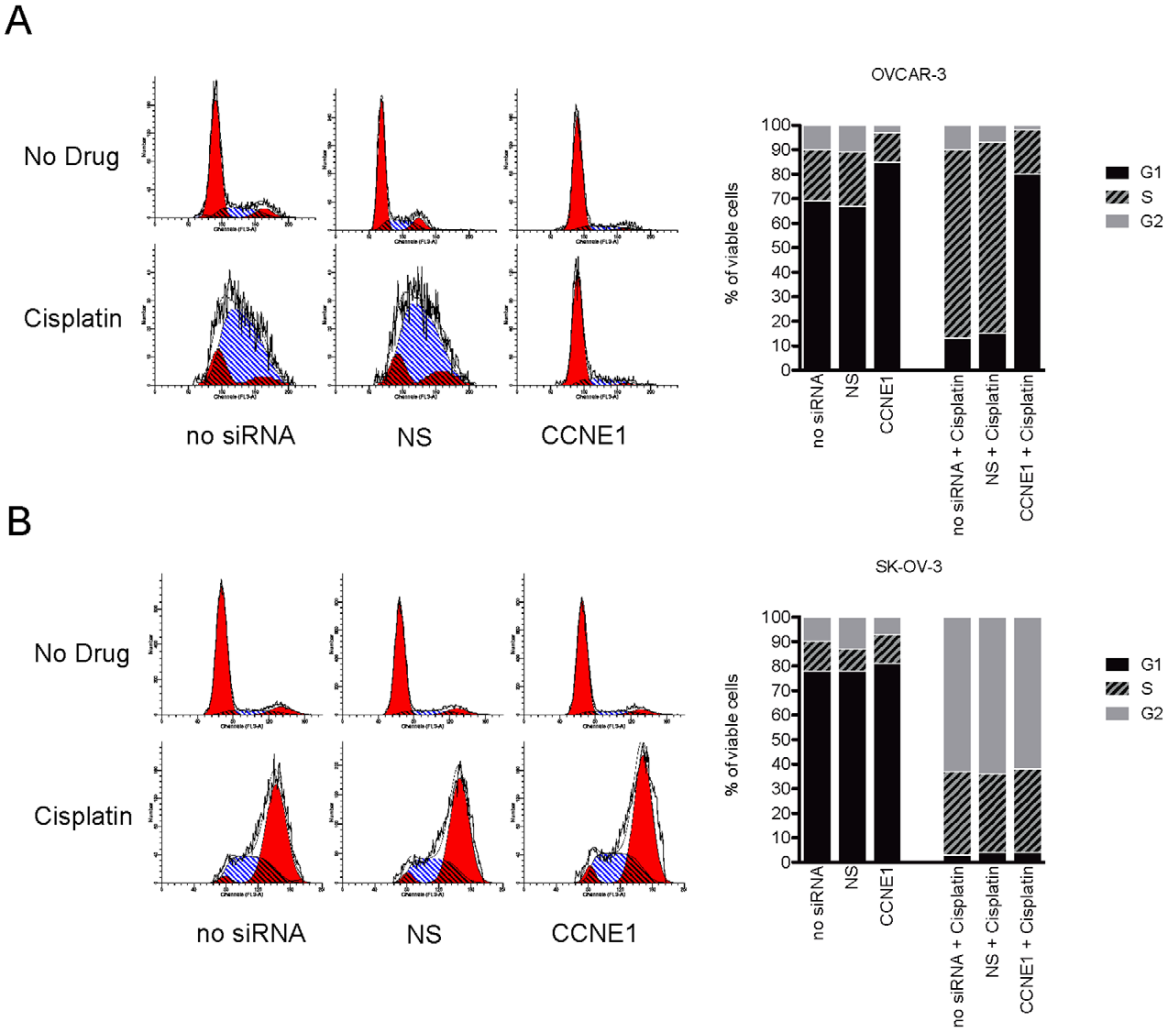
Cell cycle distribution after *CCNE1* knockdown and cisplatin treatment. (A) OVCAR-3 cells and (B) SK-OV-3 cell cycle profile (left) and proportion of cells in G1, S or G2 phase (right) for PI stained cells analyzed by flow cytometry 72 hours after transfection with *CCNE1* or NS siRNA with or without cisplatin treatment (3 µM or 6 µM for OVCAR-3 and SK-OV-3 cells respectively).

To investigate the cisplatin resistant phenotype observed in OVCAR-3 cells with *CCNE1* knockdown, we used flow cytometry to analyze cell cycle distribution following cisplatin treatment. Cisplatin treatment of OVCAR-3 resulted in a prolonged S-phase (Figure 4A) whereas SK-OV-3 cells arrested predominately in the G2 phase of the cell cycle (Figure 4B). Consistent with the requirement for Cyclin E1 in the G1/S transition [18], *CCNE1* siRNA knockdown induced G1 arrest in OVCAR-3, most evident in the presence of cisplatin (Figure 4A). By contrast, the cell cycle distribution of SK-OV-3 was unaltered by inhibition of *CCNE1* expression with or without cisplatin (Figure 4B). These observations were consistent in *CCNE1* amplified Kuramochi and OVCAR-4 cells (Figure S3). Partial G1 arrest was also observed in 19q12 unamplified IGROV-1 cells after *CCNE1* knockdown, however only in response to cisplatin treatment. As observed in the viability assay, the *CCNE1* amplified but low-expressing OVCAR-8 cells behaved similarly to control lines. We therefore concluded that the dependency of OVCAR-3, Kuramochi, and OVCAR-4 on high *CCNE1* expression resulted in a cell cycle arrest after gene knockdown, including in the presence of cisplatin, most likely accounting for the apparent cisplatin resistance observed in short-term viability assays.

**Figure 5:**
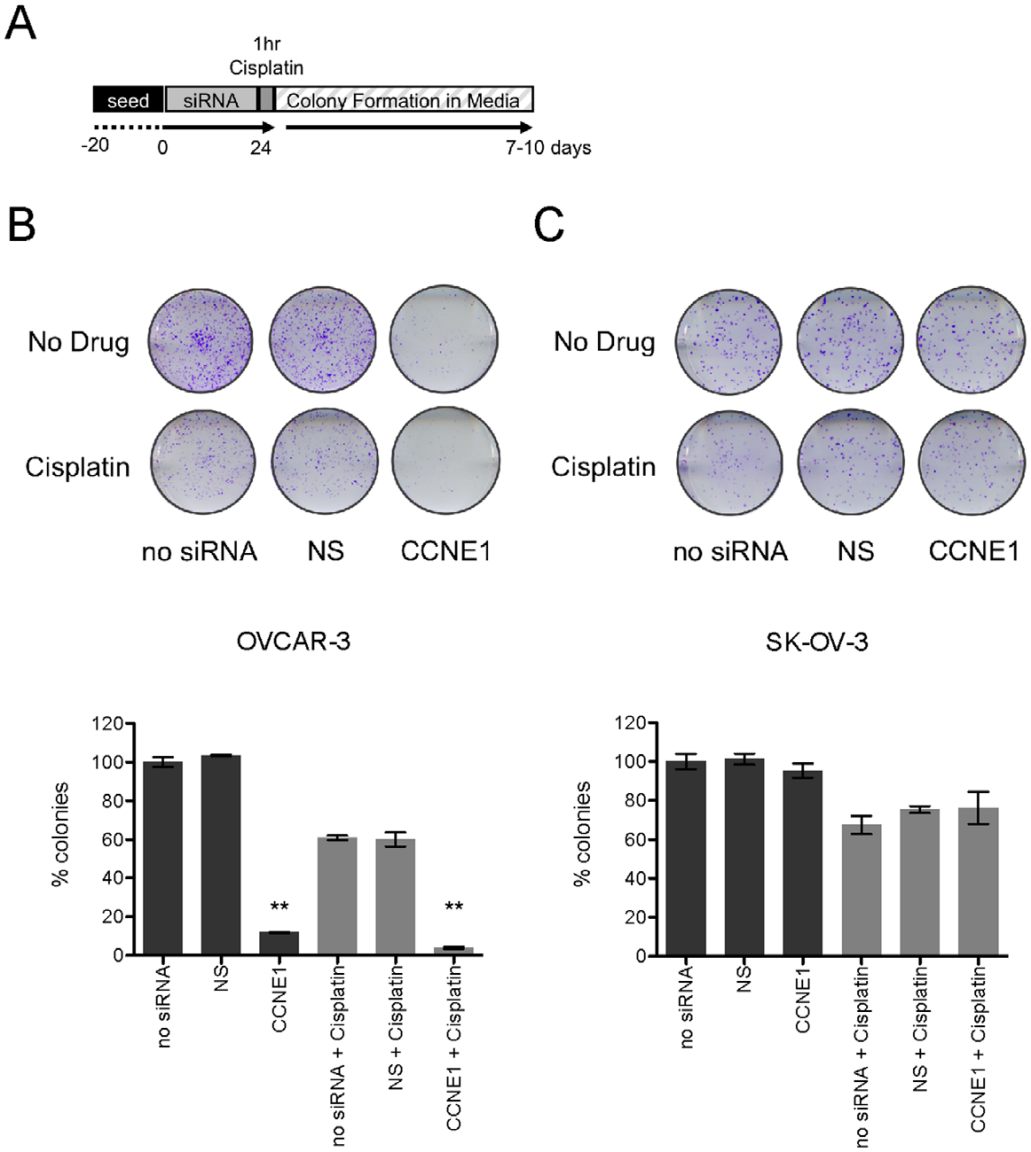
Clonogenic survival after *CCNE1* knockdown and cisplatin treatment. (A) Experimental time-course for clonogenic survival assay after siRNA transfection and cisplatin treatment. Representative crystal violet stained (B) SK-OV-3 and (C) OVCAR-3 colonies (top) and average proportion of discrete colonies formed (bottom) compared to control cells without siRNA or 1 hour cisplatin treatment (3 µM or 6 µM for OVCAR-3 and SK-OV-3 cells respectively). Error bars indicate SEM (n = 3), **p-value <0.001.

To understand the longer-term impact of *CCNE1* depletion on cell survival after cisplatin treatment we assayed the clonogenicity of lines with and without amplification of the 19q12 locus (see schematic Figure 5A). *CCNE1* knockdown profoundly reduced the clonogenic capacity of OVCAR-3 but not SK-OV-3 in the absence of drug (Figure 5B and 5C). In contrast to increased resistance to cisplatin in short-term cytotoxicity assays, *CCNE1* attenuation reduced clonogenic survival of OVCAR-3 cells after cisplatin treatment (Figure 5B). We did not explore the *in vivo* effects of *CCNE1* knockdown in ovarian tumor lines with 19q12 amplification, as we were unable to generate viable lines with stable lentivirus integration of short hairpin RNA (shRNA) directed to *CCNE1* (data not shown).

### *CCNE1* amplification is predictive of poor outcome in primary tumors

*CCNE1* copy number was measured in 43 primary ovarian tumors from patients with advanced-stage, serous invasive disease. In addition, we included data from 52 tumors from our previous genomic analysis of platinum-resistance in ovarian cancer [3] and obtained matching gene expression data for all samples [17]. All patients underwent primary surgery followed by platinum-based chemotherapy. Clinical information used to correlate *CCNE1* status with survival had over two years of additional accumulated patient follow-up data (as of June 2010) from our earlier studies.

**Figure 6:**
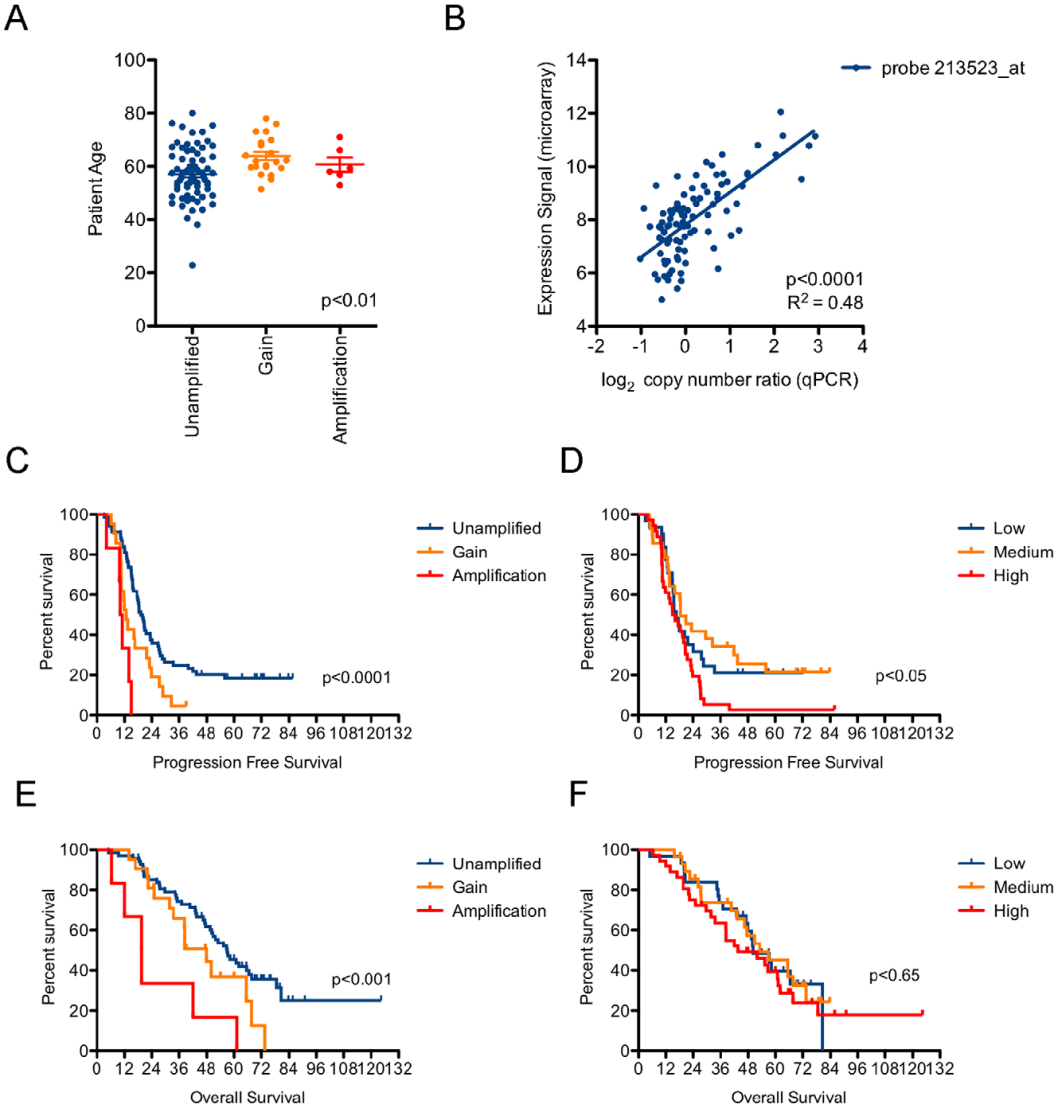
*CCNE1* copy number and gene expression associated with patient outcome. (A) Patient age distribution stratified by *CCNE1* amplification status. Kruskal-Wallis p-value reported, bars indicate mean and SEM. (B) Correlation between *CCNE1* copy number by qPCR and gene expression signal by microarray. (C) Kaplan-meier analysis of *CCNE1* unamplified (n = 68), gained (n = 21) and amplified (n = 6) ovarian cancer patients and (D) *CCNE1* low (n = 31), medium (n = 28) and high (n = 36) expressing samples for progression-free survival and (E & F) overall survival. Log-rank test p-values reported. Stratification by *CCNE1* copy number or expression status described in Materials and Methods.

Patients were stratified based on *CCNE1* copy number status as assessed by qPCR (see Materials and Methods). No difference was noted between clinical characteristics of each group apart from age, with younger patients over-represented in the *CCNE1* unamplified group (Table 1 and Figure 6A). *CCNE1* gene expression showed a strong correlation with copy number (Figure 6B) and both were correlated with progression-free survival (PFS) (Figure 6C & D). *CCNE1* copy number, but not gene expression, was also associated with overall-survival (OS) (Figure 6E & F). The most significant correlation observed was degree of *CCNE1* gain and PFS (Figure 6C), such that all cases with high-level amplification showed progressive disease within approximately twelve months from diagnosis (mean PFS of 10.7 months; Table 1).

**Table 1:** Patient characteristics by *CCNE1* copy number status.

		*CCNE1* Unamplified	*CCNE1* Gain	*CCNE1* Amplification	p-value
**Age**					
	Mean	57	64	61	<0.01^a^
	Standard Deviation	10	7	7	
	Range	23–80	52–78	53–71	
**Stage**					
	III	63 (93%)	21 (100%)	6 (100%)	0.53^b^
	IV	5 (7%)	0 (0%)	0 (0%)	
**Grade**					
	Low (well differentiated)	2 (3%)	0 (0%)	0 (0%)	0.81^b^
	Medium	30 (44%)	7 (33%)	3 (50%)	
	High (poorly differentiated)	36 (53%)	14 (67%)	3 (50%)	
**Residual Disease**					
	≤1 cm	47 (69%)	16 (76%)	3 (50%)	0.12^b^
	>1 cm	18 (26%)	2 (10%)	3 (50%)	
	Unknown	3 (4%)	3 (14%)	0 (0%)	
**Primary Treatment**					
	Pt-based	5 (7%)	1 (5%)	(0%)	1.0^b^
	Pt-based + Other	63 (93%)	20 (95%)	6 (100%)	
**PFS**					
	**(from Surgery)**				
	Mean (months)	18.91	15.72	10.68	<0.0001^c^
	Standard Deviation	10.24	7.73	3.79	
	Events	54 (79%)	20 (95%)	6 (100%)	
**OS**					
	**(from Surgery)**				
	Mean (years)	3.49	3.25	2.23	<0.001^c^
	Standard Deviation	1.56	1.55	1.73	
	Events	42 (62%)	15 (71%)	6 (100%)	
**Total Cases**		**68**	**21**	**6**	**85**

a Kruskal-Wallis,

b Fisher Exact and.

c Log-rank test p-values reported.

### Amplification of 19q12 is correlated with gain at 20q11

**Figure 7:**
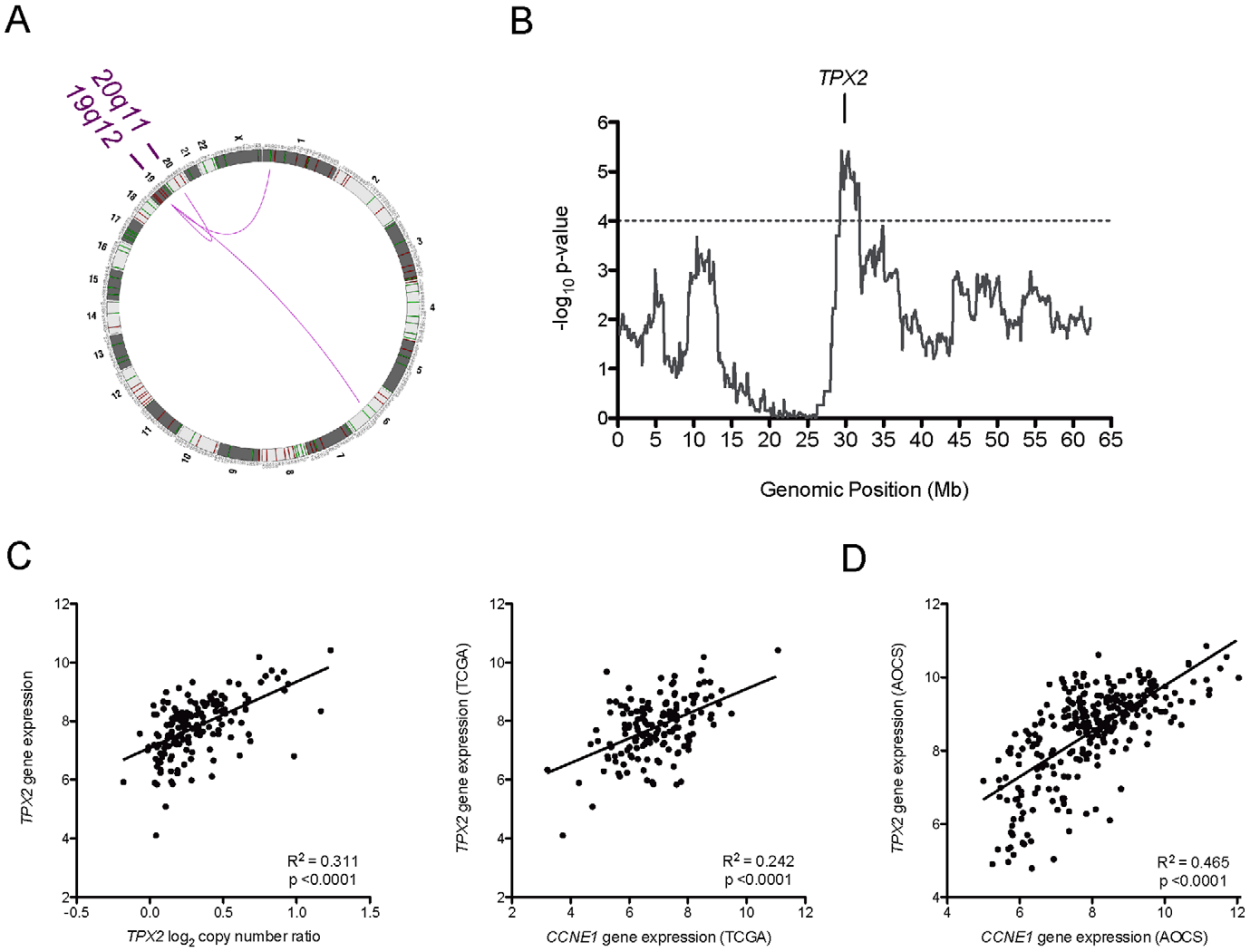
Regions of copy number change and *TPX2* gene expression associated with *CCNE1* amplification. (A) Circos plot showing regions of copy number change significantly correlated to *CCNE1* amplification (p-value <1×10^−5^) (B) Significance of copy number correlation to *CCNE1* amplification across chromosome 20. Significance threshold used to define correlation peak boundaries indicated by dotted line. (C) *TPX2* expression correlation with locus copy number and *CCNE1* expression (source: TCGA). (D) Correlation of *CCNE1* and *TPX2* gene expression in an independent data set (Tothill et al., 2009).

Having identified *CCNE1* as a critical driver within the 19q12 amplicon in ovarian cancer, we reasoned that other mutations elsewhere in the genome might interact with Cyclin E1 or be associated with drug resistance. For example, mutations that enhance the effect of or allow tumors to tolerate *CCNE1* over-expression may co-occur with 19q12 gain. We obtained both SNP-based copy number and gene expression data on 157 high-grade serous invasive tumors from The Cancer Genome Atlas Project (TCGA) for analysis. Firstly, we examined gene expression of genes whose protein products are required for processing of Cyclin E1 to active low molecular weight forms (*ELA2* and *CAPN2*) [19], [20] or its degradation (*FBXW7*) [18]. However, no statistically significant positive correlation between candidate gene expression and *CCNE1* status was observed (data not shown). We then correlated 19q12 gain with all other gains and losses within 0.1 Mb segments of the genome (see Methods S1). The top three correlated regions of copy number change were on 20q11, 1p36 and 6q27 (Figure 7A). The most significant associated gain was localized to a 2.5Mb region on chromosome 20 (Figure 7B) and has been validated in a separate unbiased analysis of genome-wide correlations of gain and loss in ovarian tumors [21].

In order to narrow gene candidates within the three regions most likely to interact with *CCNE1*, we next correlated the expression of genes within each region with *CCNE1* expression (Table 2). Expression of *TPX2* was most significantly associated with *CCNE1* expression, and was also correlated to its own amplification status (Figure 7C). The relationship between *CCNE1* and *TPX2* gene expression was further validated in a second independent data set (Figure 7D). The strong association between *TPX2* and *CCNE1* amplification and expression was intriguing given that TPX2 is a centromeric protein required for mitotic spindle function during cell division [22].

**Table 2:** 19q12 co-amplified regions of copy number change.

Region (Hg18)	Correlation Coefficient	Minimump-value	Total Genes In Region*	Candidate Genes**
20q11 (29.3–31.8 Mb)	0.360	3.7×10^−6^	13	***TPX2*** *, ASXL1, PXMP4, NECAB3, CDK5RAP1, MAPRE1, CBFA2T2, BCL2L1, PLAGL2, KIF3B, POFUT1, TM9SF4*
1p36 (23.6–26.8 Mb)	0.347	8.6×10^−6^	20	*STMN1, SH3BGRL3, HMGN2, HMGCL, CLIC4*
6q27 (166.8–170.7 Mb)	0.336	1.7×10^−5^	7	*PHF10,PSMB1*

*genes represented on hthgu133a array by ≥1 probe.

**sub-set of genes with expression correlated with *CCNE1*.

### 20q11 amplification renders cells resistant to TPX2 knockdown

**Figure 8:**
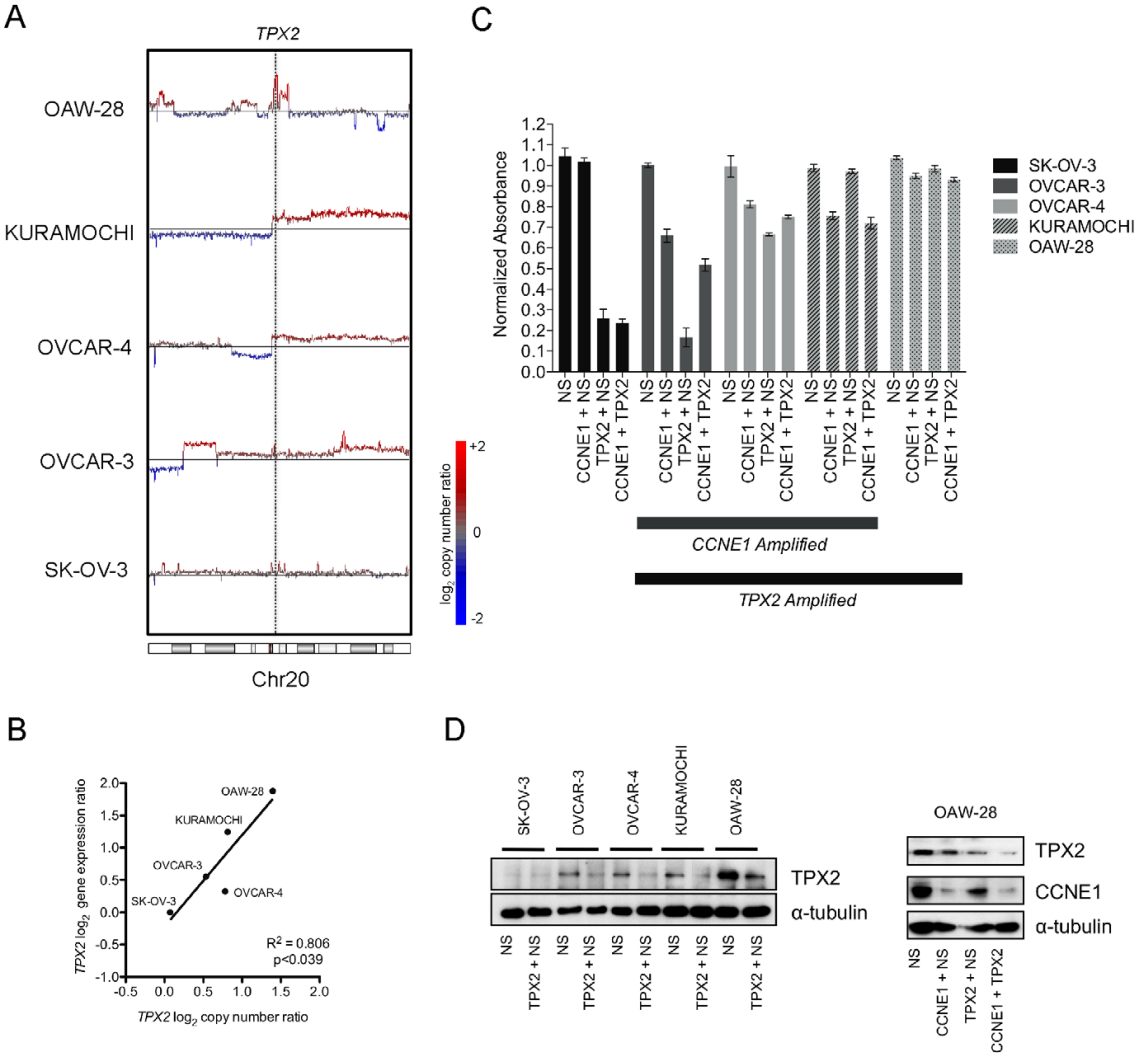
Functional analysis of *TPX2* and *CCNE1* co-amplification in ovarian tumor cell lines. (A) Affymetrix SNP 6.0 mapping microarray copy number of chromosome 20 in ovarian tumor cell lines indicating location of *TPX2*. (B) Correlation between *TPX2* copy number and gene expression by qPCR in cell lines. (C) Cell viability depicted as normalized MTS absorbance (490 nm) from three independent assays to cells treated with no siRNA in siRNA double knockdown experiments. Average normalized absorbance from MTS assay and SEM plotted (n = 3). (E) TPX2 protein expression by western blot to confirm siRNA-mediated knockdown at experimental endpoint for cell lines and CCNE1 in OAW-28 cells.

To test whether there was a functional dependence on *TPX2* in cell lines with amplification of the 20q11 locus and whether it interacts with *CCNE1*, we assessed *TPX2* status in lines used for our knockdown experiments. *TPX2* was amplified (Figure 8A) and over-expressed (Figure 8B) in all three *CCNE1* amplified and over-expressing cell lines (OVCAR-3, OVCAR-4 and Kuramochi) compared to the control line, SK-OV-3. In addition, we further identified OAW-28 as having high-level 20q11 amplification (Figure 8A, data from Sanger Cancer Genome Project Archive) and the highest level of gene expression across the tested cell lines (Figure 8B).

In contrast to the close relationship between *CCNE1* amplification and cellular sensitivity to gene knockdown, an inverse relationship was observed between *TPX2* copy number and the effects of *TPX2* siRNA (Figure 8C). For example, SK-OV-3 cells with low gene expression (Figure 8B) and minimal detectable TPX2 protein (Figure 8D), were highly sensitive to gene knockdown, whereas OAW-28 cells were essentially resistant. RT-PCR (Figure S4) and western blot (Figure 8D) analyses demonstrated efficient knockdown of *TPX2*, however protein was still detectable in some cell lines that initially had high levels of TPX2 protein. We also observed that knockdown of *CCNE1* resulted in diminished *TPX2* gene expression in 19q12 amplified lines (Figure S4 A) suggestive that *TPX2* expression is affected downstream of Cyclin E1.

Given the correlation between *CCNE1* and *TPX2* amplification, we also examined whether concurrent knockdown of both genes would further diminish viability in cell lines containing both amplifications (Figure 8C). After we allowed for a competitive effect of combining siRNAs (see Methods) no obvious interaction was observed with simultaneous knockdown of *TPX2* and *CCNE1*. Knockdown of *CCNE1* had minimal effect on OAW-28 cell viability despite efficient protein reduction in double knockdown experiments (Figure 8D). This finding is consistent with our initial results, as OAW-28 cells do not have 19q12 amplification (see Figure S1 for OAW-28 copy number at this locus).

## Discussion

We performed the first systematic siRNA knockdown of all genes within the minimally defined 19q12 amplicon in ovarian cancer showing that *CCNE1* is the key oncogenic target. Given known roles of Cyclin E1 in cancer, including de-regulation of the cell cycle and promoting genomic instability, it was the likely driver of the 19q12 locus, however other genes had not been excluded. For example, *C19orf2*, which is immediately adjacent to *CCNE1*, has recently been annotated to encode URI, an unconventional prefoldin protein. Studies of the *C. elegans* URI homologue suggest an involvement in chromatin remodeling, preventing and/or repairing endogenous genotoxic DNA damage and maintenance of genome integrity [23]. More recently, URI has been identified as a key inhibitor of protein phosphatase 1γ (PP1γ) and is involved in regulation of the mTOR/S6K1 survival pathway based on nutrient and growth factor availability [24]. Despite these intriguing biological associations, we found no evidence of URI as a driver of the 19q12 locus.

Reduction in cell viability after *CCNE1* knockdown was specific to cell lines with 19q12 amplification, with limited or no effect in non-amplified lines, indicating an ‘addiction’ [25] to *CCNE1* deregulation. Our findings validate a recent report showing amplification-specific sensitivity to *CCNE1* attenuation in OVCAR-3 and IOSE-29 cells [26]. Unamplified lines appeared to bypass siRNA mediated G1/S checkpoint arrest and apoptosis, possibly reflecting *CCNE1* independent mechanisms of cell cycle de-regulation and distinct oncogenic processes. Interestingly, increased expression of CCNE1 has recently been shown in serous tubal intraepithelial carcinoma (STIC), a proposed precursor for high-grade serous carcinoma [27]. This finding reinforces the significance of *CCNE1* de-regulation in ovarian cancer and suggests it is an early requirement in tumor evolution.

19q12 amplification is strongly associated with primary treatment failure in ovarian tumors [3] and is therefore both a potential prognostic marker and therapeutic target. Having identified *CCNE1* as the driver of 19q12 amplification we sought to understand how it contributes to primary treatment failure. In short-term cytotoxicity assays, over-expression of Cyclin E1 increases sensitivity to cisplatin [12]. Consistent with this data, we found that siRNA-mediated knockdown of *CCNE1* induced G1 arrest in 19q12-amplified cell lines, possibly protecting cells from cisplatin damage occurring through cell cycle progression. These findings, together with the lack of correlation of amplification status with *in vitro* cisplatin sensitivity of cell lines (Table S1) are at odds with the clinical behavior of primary tumors. Significantly, we found that knockdown of *CCNE1* profoundly inhibited clonogenic cell survival and this effect was augmented by cisplatin treatment (Figure 5B & C). Collectively, our findings suggest that 19q12 amplification does not increase resistance to chemotherapy *per se* but rather may confer a survival advantage post-treatment. In addition to increased cellular division, high *CCNE1* expression may assist re-entry into cell cycle from quiescence in surviving cells after chemotherapy [28]. Uniquely amongst cell cycle proteins in Drosophila, Cyclin E1 over-expression has been shown to promote self-renewal of neuroblasts [29] and may translate to an increased clonogenic capacity in tumor cells. Although we were unable to generate stable OVCAR-3 with integrated shRNA directed to *CCNE1* to further validate these findings *in vivo*, these observations suggest that *CCNE1* amplification may enhance the ability of tumor cells to repopulate the tumor after the cessation of chemotherapy.

We also sought to identify other mutational events that may enhance the effect of *CCNE1* deregulation by seeking regions of copy number change that correlate with 19q12 gain. Previous investigations of correlated gains include a recent study in breast cancer [30] where co-amplification of 8p12 and 11q13 was identified and thought to cooperate functionally through activation of independent oncogenic pathways. Cooperative networks in glioblastoma that are associated with outcome have also been identified through analysis of co-occurring copy number changes [31]. We identified copy number change of three loci at 20q11, 1p36 and 6q27 to be significantly associated with *CCNE1* amplification in ovarian tumors.

The 20q11 region contains twelve candidate genes, including *TPX2*, *ASXL1* and *BCL2L1* with expression correlated to *CCNE1* (Table 2). TPX2 is a microtubule-associated protein downstream of Ran-GTP that triggers microtubule nucleation. It both activates and is a substrate for Aurora-A kinase and is important in mitotic spindle formation and chromosome segregation during cell division [reviewed in 22]. Low copy number gain and protein over-expression has been observed in other tumor types including pancreatic tumors where siRNA knockdown reduced cell growth *in vitro*, induced apoptosis and sensitized pancreatic cell lines to paclitaxel treatment [32].

We chose to further investigate the role of *TPX2* in ovarian cancer given its expression was most significantly correlated with *CCNE1*. Furthermore, TPX2 has a plausible biological association with Cyclin E1, both having known cell cycle functions. Unlike *CCNE1* however, we did not find evidence of oncogene ‘addiction’ to *TPX2* in cells with amplification. In addition, cells sensitive to *CCNE1* knockdown showed no further reduction in viability when simultaneously treated with siRNA against *TPX2*. Notably, both OVCAR-3 and OVCAR-4 cells showed the greatest reduction in viability after single *TPX2* knockdown. Gene expression analysis in 19q12 amplified cells show that *TPX2* gene expression is reduced after *CCNE1* knockdown (Figure S4) implying *TPX2* acts downstream of *CCNE1*, and that simultaneous *TPX2* knockdown has only a minimal additive effect. The relationship between *CCNE1* and *TPX2* expression *in vitro* is consistent with the association identified in primary tumors and although our results suggest that *TPX2* acts down stream of *CCNE1*, it may not be a key driver of the 20q11 amplicon. A systematic analysis of other genes within the amplicon is required, which is often broad and contains a number of potentially important gene targets. For example, the apoptotic regulator *BCL2L1*, adjacent to *TPX2*, has been previously suggested as a 20q11 amplification target in cancer [13]. Although not correlated with *CCNE1* expression in our analysis, co-amplification of *ID1* with *CCNE1* may further contribute to cell cycle de-regulation in ovarian cancer. ID1 is involved in proliferation and differentiation, and functions by inhibiting binding and activity of other helix-loop-helix transcription factors. In breast cancer cells, gene knockdown has been shown to decrease *CCNE1* expression and Cyclin E1/CDK2 activity [33]. Interestingly, we also note the presence of a non-coding RNA (NCRNA00028) and two micro-RNAs (hsa-mir-1825 and hsa-mir-3193) within the defined region of chromosome 20q11 gain. The potential effect of amplification on their function or subsequent de-regulation of downstream targets was not investigated in our current analysis. However, we are not aware of any evidence that would link these molecules to the regulation of *CCNE1*.

In primary tumors, *CCNE1* copy number was significantly associated with shorter progression-free and overall survival, with tumors bearing high-level amplification showing the worst outcome (Figure 6). Copy number status was more informative than gene expression; a significant correlation with high expression and PFS was observed, but not for OS. Similar to *ERBB2* amplification in breast cancer, high-level *CCNE1* amplification may therefore have predominant clinical utility in identifying patients most likely to have a poor response to standard treatment. Interestingly, younger patients were less likely to have *CCNE1* amplified tumors (Table 1 and Figure 6A) suggesting a difference between the etiology of these tumors.

Our observations have potential implications for future therapeutic strategies based on targeting of cell cycle deregulation such as via cyclin-dependent kinase (CDK) inhibition. To date, the clinical success of single-agent CDK inhibitors has been disappointing [34]. Limited success may in part relate to off-target drug effects or redundancy between CDKs. It is anticipated that chemotherapeutic agents may sensitize cells to CDK inhibition [35]. However as shown here, interruption of G1/S transition may increase resistance to G1/S-phase targeted treatment and therefore *CCNE1/CDK2* targeted therapies may need to appropriately phased with conventional therapy. Additionally, the surprising degree of resistance of cells without 19q12 amplification to *CCNE1* knockdown suggests that CDK2 inhibitor clinical trials should incorporate careful pre-selection of ovarian cancer patients and other tumor subtypes associated with Cyclin E1 over-expression, such as triple-negative and basal-like breast tumors [36].

## Materials and Methods

### Ethics Statement

This study was approved by the Human Research Ethics Committees at the Peter MacCallum Cancer Centre, Queensland Institute of Medical Research, University of Melbourne and all participating hospitals. Written informed consent was obtained from all participants in this study.

### Cell Lines

Ovarian cell lines were maintained at 37° and 5% CO_2_ in RPMI 1640 containing 10% (v/v) FCS, 50 U.mL^−1^ penicillin and 50 ug.mL^−1^ streptomycin, except for OAW-28 cells which were maintained in DMEM containing 10% (v/v) FCS, 50 U.mL^−1^ penicillin, 50 ug.mL^−1^ streptomycin and 0.2 U.mL^−1^ insulin. Transfection and drug-treatment assays were performed in antibiotic-free medium.

### Copy Number Data

Microarray data was obtained from Tumorscape (www.broadinstitute.org/tumorscape), the Cancer Genome Atlas Project (TCGA) (cancergenome.nih.gov) for primary tumors and the Sanger Cancer Genome Project Archive (http://www.sanger.ac.uk/genetics/CGP/Archive/) for ovarian cell lines. Data was visualized using Partek Genomics Suite 6.4 (Partek Inc., St Louis, MO) and Tree View [37]. Further copy number analysis is described in Methods S1.

### Molecular Methods

Cell line DNA was extracted using a DNeasy Kit (Qiagen, Valencia, CA) and quantitative-PCR (qPCR) analysis of *CCNE1* DNA copy number status was performed as described previously [3]. Total RNA was extracted from cell pellets using an RNeasy mini Kit (Qiagen) and reverse transcribed using M-MLV prior to SYBR green qPCR as detailed in Methods S1. Primer sequences to measure gene expression were obtained from qPrimerDepot [38]. Two primer sets were used to assess *TPX2* copy number and were designed using Primer3 [39] or obtained elsewhere [32]. All primers are listed in Table S2.

### siRNA Transfection

Cells were seeded at a density of 5–10×10^3^ cells per well in 96 well plates (for viability assays), Lab-Tek II Chamber Slides (Sigma Aldrich, St Louis, MO) (for TUNEL staining) or 3–6×10^4^ cells in 24 well plates (for all other assays) approximately 20 hours prior to transfection. Selected seeding densities resulted in cell growth as a sub-confluent monolayer at transfection. ON-Target plus siRNA pools (listed in Table S3) and transfection reagents were obtained from Dharmacon (ThermoFisher Scientific, Lafayette, CO). Optimal transfection conditions were determined based on maximal gene knockdown and minimal cytotoxicity as assessed by RT-PCR and MTS cell viability assay (see Methods S1). Nuclear localization of siGLO Green RNA duplex was used to monitor transfection efficiency by fluorescence microscopy 24–48 hours after transfection (data not shown). Transfection reagent and siRNA pools were pre-incubated at room temperature for 15 minutes in serum-free media (20% of final transfection volume) to facilitate formation of lipid-siRNA complexes. Cells were then incubated in final transfection mix containing 0.4% (v/v) DharmaFECT 2 and 50 nM siRNA in antibiotic-free media with serum. In double knockdown experiments, total siRNA amount was normalized between treatment groups by addition of a non-silencing siRNA up to 100 nM. After 24 hours, media was replaced and following a further 72 hours, cells assayed for viability, cisplatin sensitivity (at a pre-determined IC50 dose, Table S4), cell cycle distribution, clonogenic survival or apoptosis as described below and in Methods S1.

### Western Blot

Whole cell protein lysates were boiled, resolved by SDS-PAGE using 12.5% (w/v) acrylamide gels and then transferred to PVDF membranes. Blots were blocked in 5% (w/v) non-fat milk powder in PBS-T (0.1% Tween 20 in PBS) and probed overnight at 4°C in 1∶500 primary antibody against human Cyclin E1 (clone HE12) (Santa Cruz Biotechnology, Santa Cruz, CA) or TPX2 (clone 18D5) (Biolegend, San Diego, CA). Membranes were washed in PBS-T and incubated with 1∶7000 dilution of peroxidase-conjugate secondary antibody for 1 hr at room temperature, washed and developed by chemoluminescence before being exposed to radiographic film. Blots were re-probed with an antibody against α-tubulin to assess protein loading.

### Flow Cytometry

Control, transfected and cisplatin treated cells were rinsed in PBS, trypsinized to form a single-cell suspension and fixed in 70% ice-cold ethanol. Cell were pelleted and resuspended in a solution containing 50 µg.mL^−1^ propidium iodide and 100 U.mL^−1^ RNAseA (Qiagen) for 30 minutes at room temperature. Up to 10,000 cells were then counted by FACS. Viable cell cycle profiles and percentage of cells in each cell cycle phase was determined using Modfit LT (Beckman Coulter, Brea, CA).

### TUNEL Staining

Apoptotic cells were identified using the ApopTag® In Situ Apoptosis Detection Kit (Intergen, Purchase, NY). A minimum of eight, uniformly spaced images were taken per well and positively and negatively stained cells counted by a researcher blinded to the experimental setup.

### Clonogenic Survival Assay

Transfected cells were treated with cisplatin for 1 hour then PBS washed, trypsinized to form a single-cell suspension, counted (Coulter Counter) and cell number equalized for each experimental condition. Cells were then seeded at low density in wells of a 6-well plate in triplicate and left to form colonies for up to ten days. The number of plated cells differed depending on cell line plating efficiency; 500 and 15,000 cells were used for SK-OV-3 and OVCAR-3 respectively. Cell colonies were then fixed and stained with 20% (v/v) methanol and 0.1% (w/v) crystal violet. Cells were rinsed in water, air-dried and discrete colonies counted using MetaMorph (Molecular Devices, Sunnyvale, CA).

### *CCNE1* Copy Number and Gene Expression in Primary Tumors

Tumor samples and clinical data were obtained from women with advanced stage, serous invasive disease enrolled through the Australian Ovarian Cancer Study (www.aocstudy.org). This project had institutional ethics review board approval at all participating centers.

Samples were segregated based on *CCNE1* copy number level as assessed by qPCR (above) using a log_2_ copy number ratio cut-off of ≥0.5 (∼3 copies) for gain and ≥2 (∼8 copies) for amplification (Table 1). Matched expression data from Affymetrix U133 plus 2.0 microarrays was obtained from a previous study [17]. The *CCNE1* probe showing the highest signal level (213523_at) was selected for our analysis and showed a significant correlation with gene copy number (Figure 6B). Samples were segregated into low, medium and high expression of *CCNE1*, where tumors with high expression were defined as those above the median signal value + [0.5 x median absolute deviation (MAD)] and low expressing tumors where those below the median signal value – [0.5 *MAD]. Statistical analysis was performed in GraphPad Prism (GraphPad Software, San Diego, CA). PFS and OS was calculated from the date of diagnosis (surgery).

## Supporting Information

Figure S1 **Heat-map of copy number change in ovarian tumor cell lines.** Affymetrix SNP 6.0 mapping microarray copy number of chromosome 19 in 22 ovarian tumor cell lines between 34–36 Mb (source: Sanger Cancer Genome Project Archive). (TIF)

Figure S2 ***CCNE1*** **gene and protein expression in knockdown experiments.** (A) Correlation between *CCNE1* copy number status and gene expression by qPCR in ovarian cell lines. (B) *CCNE1* gene expression in ovarian cell lines normalized to SK-OV-3 with no siRNA treatment after transfection with *CCNE1* or non-silencing siRNA. (C) CCNE1 protein expression by western-blot to confirm siRNA-mediated Cyclin E1 knockdown at experimental endpoint in ovarian cell lines. (TIF)

Figure S3 **Cell cycle distribution after** ***CCNE1*** **knockdown and cisplatin treatment in additional cell lines.** Cycle profile (left) and proportion of cells in G1, S or G2 phase (right) for PI stained cells analyzed by flow cytometry after transfection with CCNE1 or non-silencing siRNA and with or without cisplatin treatment in (A) IGROV-1, (B) OVCAR-8, (C) Kuramochi and (D) OVCAR-4 cell lines. (TIF)

Figure S4 ***CCNE1*** **and** ***TPX2*** **gene expression in combined knockdown experiments.** *CCNE1* and *TPX2* gene expression ratios in ovarian cell lines normalized to no siRNA treated cells in each line after single or combined transfection with *NS*, *CCNE1* and *TPX2* siRNA. (TIF)

Table S1 **Cisplatin IC50 values from 72 hour cytotoxicity assays.** (DOC)

Table S2 **Primer Sequences.** (DOC)

Table S3 **ON-Target plus siRNA pools (Dharmacon).** (DOC)

Table S4 **Cisplatin experimental doses and effect on cell viability.** (DOC)

Methods S1 **Supplementary Methods.** (DOC)
