# Supplementary figures and images for: Amplicon-Dependent CCNE1 Expression Is Critical for Clonogenic Survival after Cisplatin Treatment and Is Correlated with 20q11 Gain in Ovarian Cancer
Source: PLoS One. 2010 Nov 12;5(11):e15498. doi: 10.1371/journal.pone.0015498 (PMC2980490; doi:10.1371/journal.pone.0015498)

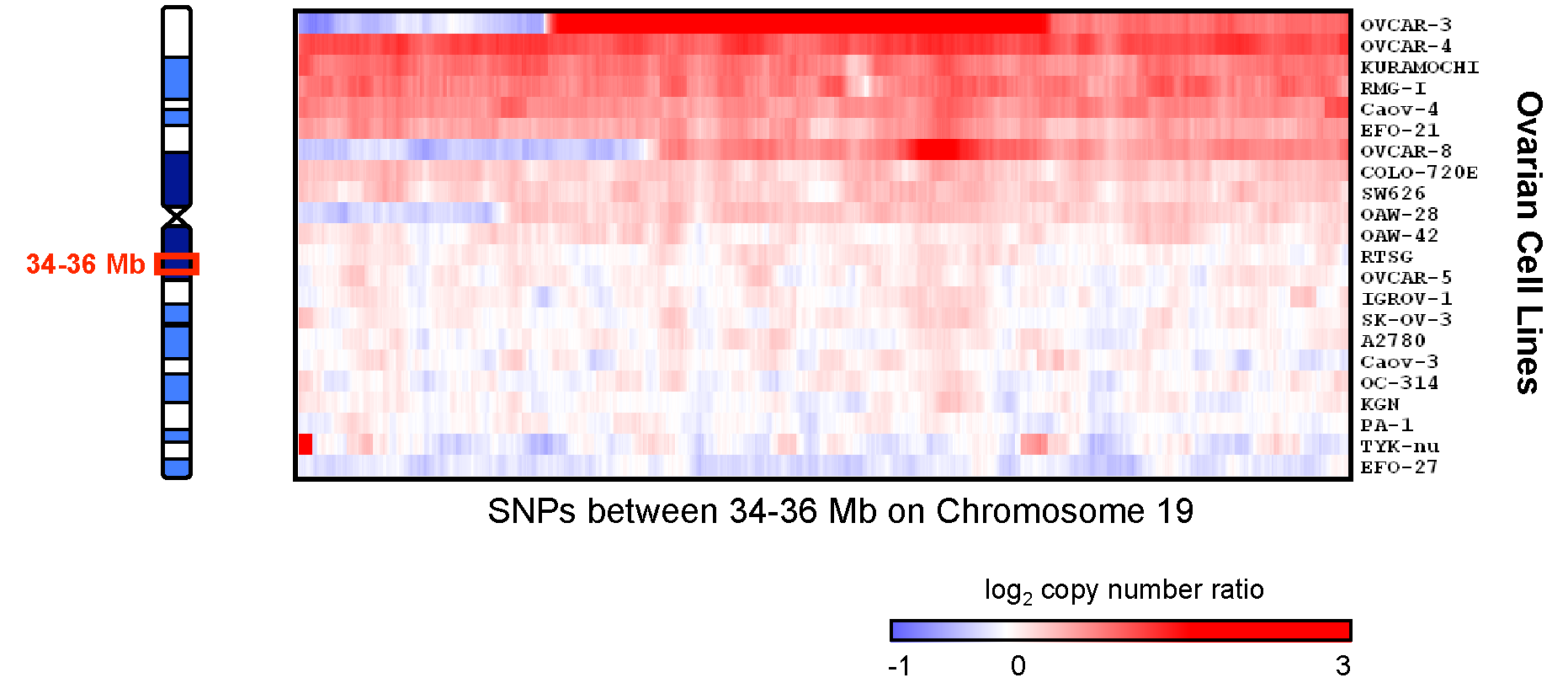

Supplement: Figure S1 — Heat-map of copy number change in ovarian tumor cell lines. Affymetrix SNP 6.0 mapping microarray copy number of chromosome 19 in 22 ovarian tumor cell lines between 34–36 Mb (source: Sanger Cancer Genome Project Archive). (TIF) [file pone.0015498.s001.tif]

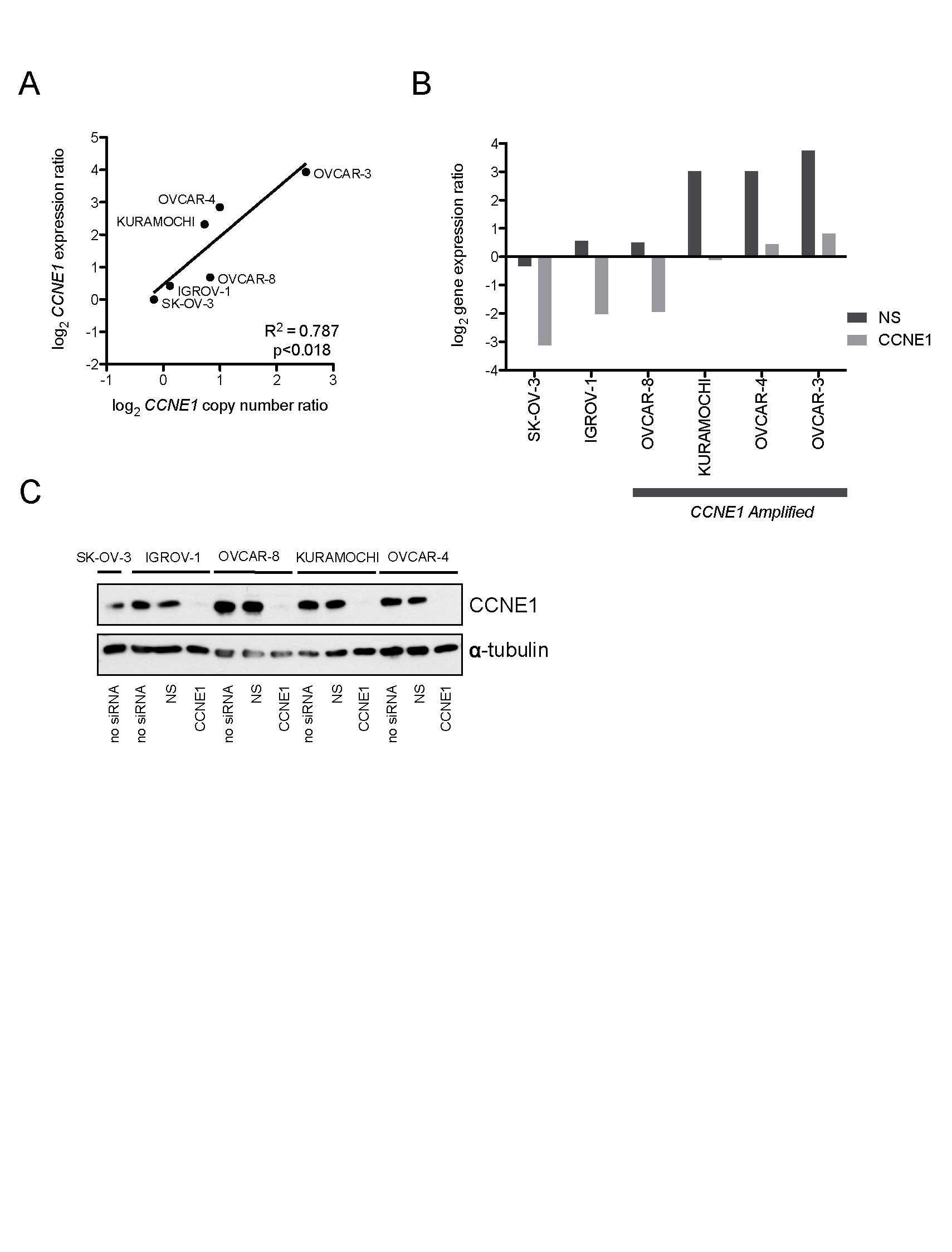

Supplement: Figure S2 — CCNE1 gene and protein expression in knockdown experiments. (A) Correlation between CCNE1 copy number status and gene expression by qPCR in ovarian cell lines. (B) CCNE1 gene expression in ovarian cell lines normalized to SK-OV-3 with no siRNA treatment after transfection with CCNE1 or non-silencing siRNA. (C) CCNE1 protein expression by western-blot to confirm siRNA-mediated Cyclin E1 knockdown at experimental endpoint in ovarian cell lines. (TIF) [file pone.0015498.s002.tif]

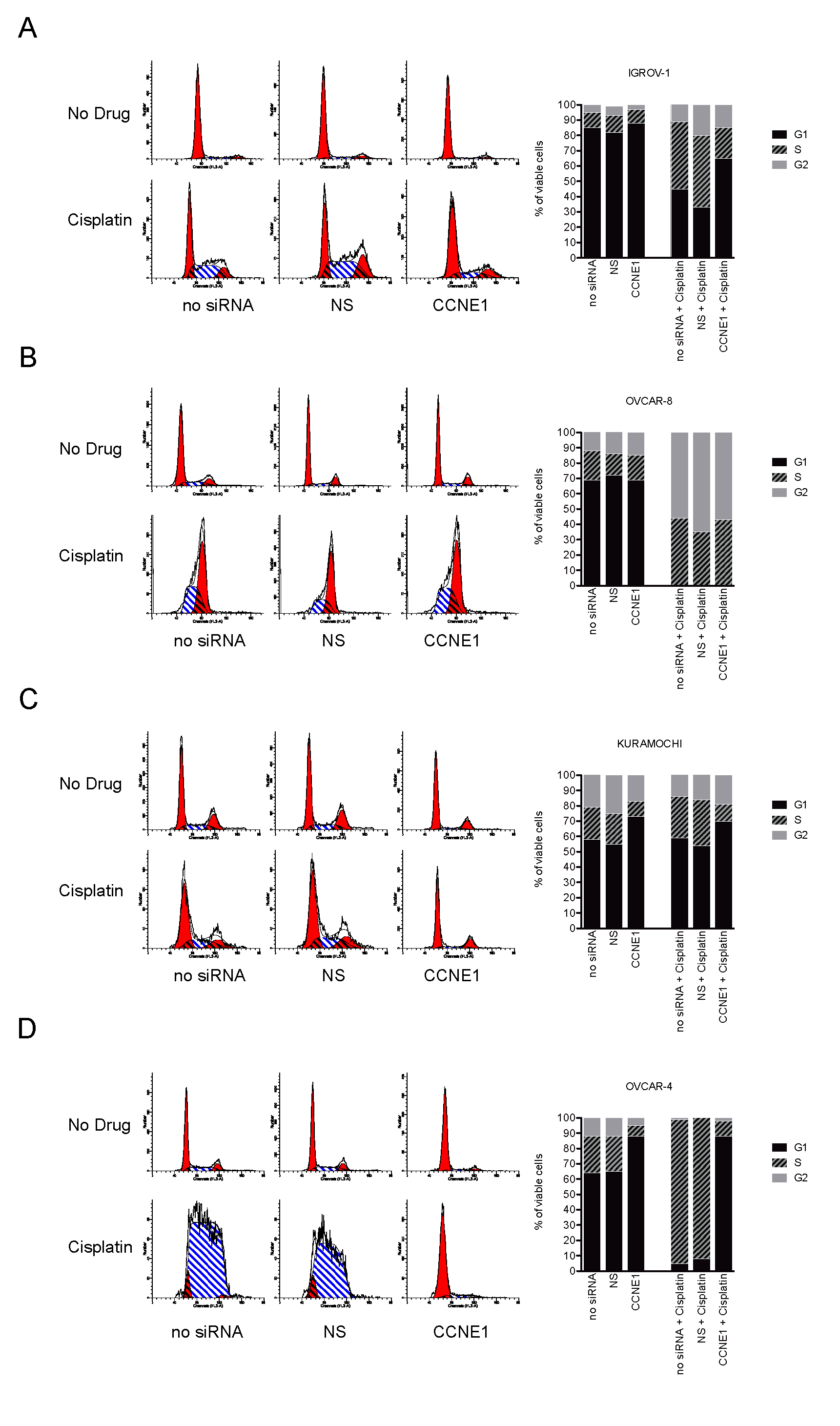

Supplement: Figure S3 — Cell cycle distribution after CCNE1 knockdown and cisplatin treatment in additional cell lines. Cycle profile (left) and proportion of cells in G1, S or G2 phase (right) for PI stained cells analyzed by flow cytometry after transfection with CCNE1 or non-silencing siRNA and with or without cisplatin treatment in (A) IGROV-1, (B) OVCAR-8, (C) Kuramochi and (D) OVCAR-4 cell lines. (TIF) [file pone.0015498.s003.tif]

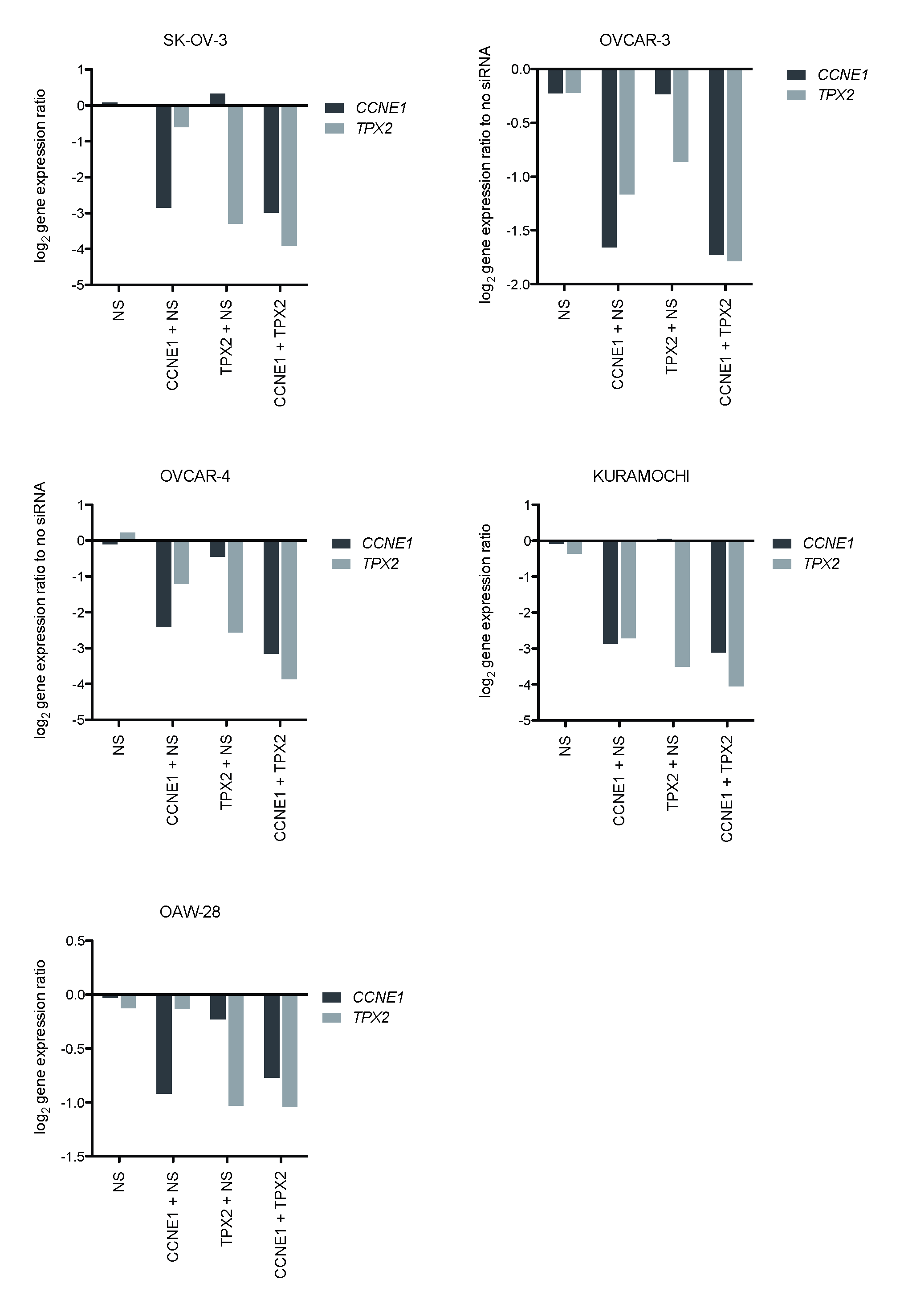

Supplement: Figure S4 — CCNE1 and TPX2 gene expression in combined knockdown experiments. CCNE1 and TPX2 gene expression ratios in ovarian cell lines normalized to no siRNA treated cells in each line after single or combined transfection with NS, CCNE1 and TPX2 siRNA. (TIF) [file pone.0015498.s004.tif]
