# Supplementary material for: Amplicon-Dependent CCNE1 Expression Is Critical for Clonogenic Survival after Cisplatin Treatment and Is Correlated with 20q11 Gain in Ovarian Cancer
Source: PLoS One. 2010 Nov 12;5(11):e15498. doi: 10.1371/journal.pone.0015498 (PMC2980490; doi:10.1371/journal.pone.0015498)
Supplement: Table S1 — Cisplatin IC50 values from 72 hour cytotoxicity assays. (DOC) [file pone.0015498.s005.doc]

**Table S1. Cisplatin IC50 values from 72 hour cytotoxicity assays.**

|  | **19q12 Status** | **IC50 (µM)** | **95% CI** |
| --- | --- | --- | --- |
| SK-OV-3 | Unamplified | 5.08 | 4.50 - 5.74 |
| IGROV-1 | Unamplified | 2.84 | 2.62 - 3.09 |
| OVCAR-8 | Amplified | 8.03 | 6.68 - 9.64 |
| KURAMOCHI | Amplified | 6.44 | 5.81-7.14 |
| OVCAR-4 | Amplified | 2.52 | 2.44 - 2.61 |
| OVCAR-3 | Amplified | 1.93 | 1.59 - 2.34 |
