# Supplementary material for: Amplicon-Dependent CCNE1 Expression Is Critical for Clonogenic Survival after Cisplatin Treatment and Is Correlated with 20q11 Gain in Ovarian Cancer
Source: PLoS One. 2010 Nov 12;5(11):e15498. doi: 10.1371/journal.pone.0015498 (PMC2980490; doi:10.1371/journal.pone.0015498)
Supplement: Table S2 — Primer Sequences. (DOC) [file pone.0015498.s006.doc]

**Table S2. Primer Sequences.**

| **Locus** | **Left Primer** | **Tm** | **Right Primer** | **Tm** | **Product Size** |
| --- | --- | --- | --- | --- | --- |
| *UQCRFS1* | TTCCTCAGCCGGGAGTC | 59.8 | CCTTGATGTCTGTGTGGGAA | 59.5 | 109 |
| *POP4* | GAAGCGGTCCGAGAATGAAG | 62.2 | GCTGTGCTCCTGAAGGCT | 59.2 | 90 |
| *PLEKHF1* | CTACTGCGGTGTGGACTCG | 60.5 | CTGGCTGTTGATCTCCGTGT | 61.3 | 102 |
| *C19orf12* | CGCGTAGACCTCCGCTC | 60.7 | TAGGATCTGAGGAACCGGC | 60.2 | 94 |
| *CCNE1* | GAAATGGCCAAAATCGACAG | 60.4 | TCTTTGTCAGGTGTGGGGA | 60.1 | 110 |
| *C19orf2* | CGCGAGGAGCAGGAAAA | 61.2 | GGCAAGGTGCTGAGTCTTTC | 60.0 | 107 |
| *ZNF536* | TCCTCGCCATCCTCCTC | 59.8 | CAGTCCTTCATGGCCGAG | 60.4 | 107 |
| *GAPDH* | AAGGTGAAGGTCGGAGTCAA | 59.7 | AATGAAGGGGTCATTGATGG | 59.6 | 108 |
| *ACTB* | GCACAGAGCCTCGCCTT | 60.2 | GTTGTCGACGACGAGCG | 60.1 | 93 |
| *HPRT1* | GTTATGGCGACCCGCAG | 61.2 | ACCCTTTCCAAATCCTCAGC | 60.4 | 107 |
| *TPX2* | AGGGGTGAAAGAGAAGATTGC | 59.7 | CCACTTTTCTCAGCAGGTCA | 59.0 | 91 |
| *gTPX2** (1) | AGGGGCCCTTTGAACTCTTA | 60.1 | TGCTCTAAACAAGCCCCATT | 59.7 | 84 |
| *gTPX2** (2) | GGAACCTTTTCAGCTGGCTA | 59.5 | CTGTAGTCTGGCCTCCTCCA | 60.4 | 115 |

*average data from two primer sets used for *TPX2* copy number assessment from genomic DNA. gTPX2(1) obtained from Warner et al., 2009.
