# Supplementary material for: Amplicon-Dependent CCNE1 Expression Is Critical for Clonogenic Survival after Cisplatin Treatment and Is Correlated with 20q11 Gain in Ovarian Cancer
Source: PLoS One. 2010 Nov 12;5(11):e15498. doi: 10.1371/journal.pone.0015498 (PMC2980490; doi:10.1371/journal.pone.0015498)
Supplement: Table S3 — ON-Target plus siRNA pools (Dharmacon). (DOC) [file pone.0015498.s007.doc]

**Table S3. ON-Target plus siRNA pools (Dharmacon).**

| **siRNA** | **Transcriptional Target** | **Catalogue Number** |
| --- | --- | --- |
| UQCRFS1 | NM_006003 | L-020100-00 |
| POP4 | NM_006627 | L-020046-00 |
| PLEKHF1 | NM_024310 | L-018423-00 |
| C19orf12 | NM_001031726; NM_031448 | L-014731-01 |
| CCNE1 | NM_001238; NM_057182 | L-003213-00 |
| C19orf2 | NM_003796; NM_134447 | L-017399-00 |
| ZNF536 | NM_014717 | L-020506-01 |
| GAPDH | NM_002046 | D-001830-10-05 |
| TPX2 | NM_012112 | L-010571-00 |
| Non-targeting Pool | N/A | D-001810-10-05 |
