# Supplementary material for: Amplicon-Dependent CCNE1 Expression Is Critical for Clonogenic Survival after Cisplatin Treatment and Is Correlated with 20q11 Gain in Ovarian Cancer
Source: PLoS One. 2010 Nov 12;5(11):e15498. doi: 10.1371/journal.pone.0015498 (PMC2980490; doi:10.1371/journal.pone.0015498)
Supplement: Table S4 — Cisplatin experimental doses and effect on cell viability. (DOC) [file pone.0015498.s008.doc]

**Table S4. Cisplatin experimental doses and effect on cell viability.**

|  | **19q12 Status** | **Treatment Dose (µM)** | **Mean Viability** | **SEM** |
| --- | --- | --- | --- | --- |
| SK-OV-3 | Unamplified | 6 | 30.9% | 4.0% |
| IGROV-1 | Unamplified | 3 | 24.4% | 3.3% |
| OVCAR-8 | Amplified | 9 | 28.8% | 4.3% |
| KURAMOCHI | Amplified | 7 | 36.3% | 5.4% |
| OVCAR-4 | Amplified | 3 | 24.6% | 4.0% |
| OVCAR-3 | Amplified | 3 | 26.9% | 4.6% |
