# Supplementary material for: Amplicon-Dependent CCNE1 Expression Is Critical for Clonogenic Survival after Cisplatin Treatment and Is Correlated with 20q11 Gain in Ovarian Cancer
Source: PLoS One. 2010 Nov 12;5(11):e15498. doi: 10.1371/journal.pone.0015498 (PMC2980490; doi:10.1371/journal.pone.0015498)
Supplement: Methods S1 — Supplementary Methods. (DOC) [file pone.0015498.s009.doc]

**Methods S1**

Reverse Transcription and qPCR

Prior to reverse transcription, 1 μg total RNA was denatured with an equal amount of random hexamer primers (Promega, Madison, WI) at 70ºC for 5 minutes then cooled on ice to anneal. Synthesis of cDNA was performed using 200U M-MLV reverse transcriptase and 12.5 nmol of each dNTP in M-MLV reaction buffer (all reagents Promega) at 42 ºC for 90 minutes followed by a 95º enzyme deactivation step for 5 minutes. Gene expression was measured by quantitative PCR (qPCR) using SYBR Green qPCR assay on the 7900HT Fast Real-Time PCR system (Applied Biosystems, Foster City, CA). PCR was performed in triplicate 10 μL reactions containing approximately 2 ng cDNA and 1 μmol of each primer plus 5 μL SYBR green master mix (Applied Biosystems). Conditions for amplification were 50°C for 2 minutes, 95°C for 10 minutes followed by 40 cycles of 95°C for 15 seconds and 60°C for 1 minute. Threshold cycle numbers were obtained using Sequence Detection Software 2.3 (Applied Biosystems) using default settings and gene expression calculated using the comparative threshold cycle method (∆∆CT) against the average Ct value obtained for two endogenous control genes (*ACTB* and *HPRT1*).

Cell Viability and Drug Sensitivity Assays

Cell viability was determined using the CellTiter 96® Aqueous Non-Radioactive Cell Proliferation Assay (Promega, Madison, WI). In the final hour of incubation, 20 μL of MTS reagent was added to each well and the amount of MTS tetrazolium salt bioreduced to formazan measured by absorbance at 490 nm. Absorbance was normalized to no siRNA control cells with transfection reagent after subtraction of background absorbance from media alone. To determine cisplatin IC50 values, cells were seeded at a density of 5 x 10^3^ cells per well in a 96-well plate and treated with cisplatin the following day for 72 hours. The IC50 dose was approximated by fitting a four-parameter dose-response curve (Hill equation) and all parameters used in curve comparisons (Figure 3B) using Prism 5 (GraphPad Software, La Jolla, CA). IC50 values ranged from between approximately 2-8 µM as shown in Table S1. Drug concentrations were chosen that were marginally higher than the IC50 value to give consistent toxic effect, while allowing for a change in cisplatin sensitivity or resistance to be observed (Table S4).

To measure change in cisplatin cytotoxicity, siRNA transfected cells were washed in PBS then treated with near IC50 doses for 72 hours. Cell viability was determined by MTS assay at the experimental endpoint. The p-value significance of each siRNA effect was calculated by t-test comparison to non-silencing siRNA transfected cells.

Correlated Regions of Copy Number Change

Affymetrix SNP 6.0 and hthgu133a gene expression data was obtained for 157 serous tumors from the Cancer Genome Atlas Project (TCGA) (cancergenome.nih.gov). All SNP CEL files were normalized in a single batch using the R package ‘aroma.affymetrix’ and then segmented using the circular binary segmentation (CBS) algorithm to improve the signal to noise ratio as described elsewhere (Gorringe et al., *in press*). Pearson’s correlation coefficient was computed between copy number level of 19q12 loci and all other genomic loci divided into 100,000 base pair segments. Three chromosomes contained SNPs with minimal significant correlation p-values less than 1 x 10^-5^ and were selected as top candidate regions. Each correlation peak was then mapped to include the maximum significant SNP with boundaries including consecutive surrounding SNPs with p-values up to one order of magnitude higher (<1 x 10^-4^) allowing for one SNP to fall above this threshold. Expression of genes within the defined peaks was then correlated with *CCNE1* levels and those genes with p-values <0.01 reported in Table 1. The correlation between *CCNE1* and *TPX2* expression was validated in an independent cohort of 215 high-grade serous tumors obtained from a previous study (Tothi*ll et a*l., 2008).
